# Ion channels of cold transduction and transmission

**DOI:** 10.1085/jgp.202313529

**Published:** 2024-07-25

**Authors:** Cheyanne M. Lewis, Theanne N. Griffith

**Affiliations:** 1 Department of Physiology and Membrane Biology, University of California Davis, Davis, CA, USA

## Abstract

Thermosensation requires the activation of a unique collection of ion channels and receptors that work in concert to transmit thermal information. It is widely accepted that transient receptor potential melastatin 8 (TRPM8) activation is required for normal cold sensing; however, recent studies have illuminated major roles for other ion channels in this important somatic sensation. In addition to TRPM8, other TRP channels have been reported to contribute to cold transduction mechanisms in diverse sensory neuron populations, with both leak- and voltage-gated channels being identified for their role in the transmission of cold signals. Whether the same channels that contribute to physiological cold sensing also mediate noxious cold signaling remains unclear; however, recent work has found a conserved role for the kainite receptor, GluK2, in noxious cold sensing across species. Additionally, cold-sensing neurons likely engage in functional crosstalk with nociceptors to give rise to cold pain. This Review will provide an update on our understanding of the relationship between various ion channels in the transduction and transmission of cold and highlight areas where further investigation is required.

## Teaser

Lewis and Griffith review developments in ion channels of cold transduction and transmission.

## Introduction

The detection of thermal stimuli is an important adaptive feature necessary for body temperature regulation and survival. The formation of a thermal percept begins with primary sensory neurons that transmit temperature information from the peripheral nervous system to the central nervous system (Vriens et al., 2014). Cold sensations are transmitted by specialized neurons of the dorsal root and trigeminal ganglia (DRG and TG, respectively), referred to as cold receptors, with either unmyelinated C fibers or thinly myelinated Aδ afferents (Lewis and Griffith, 2022; McKemy, 2013, 2018). In vitro, ex vivo, and in vivo electrophysiology and imaging studies have characterized cold-sensing neurons based on their firing properties (Viana et al., 2002), temperature thresholds (Darian-Smith et al., 1979; McKemy, 2013; Yarmolinsky et al., 2016), and size (Vriens et al., 2014), highlighting a functional diversity in cold receptor signaling that is not well understood. Although our knowledge of the ion channels that give rise to this diversity is expanding, many unanswered questions remain.

The transformation of a cold stimulus into an electrical signal relies on temperature-sensitive transient receptor potential (TRP) channel activation and voltage-gated ion channel activity (Voets, 2012). Currently, transient receptor potential melastatin 8 (TRPM8) is the only bona fide mammalian innocuous cold transducer (McKemy et al., 2002; Peier et al., 2002). On the other hand, a variety of voltage-gated channels have been reported to regulate electrical signaling in cold receptors. Channels of the K_V_1 family, such as K_V_1.1 and K_V_1.2, regulate action potential threshold and firing kinetics, and K_V_1 channel density can determine the activation threshold of cold-sensing DRG and TG neurons (Viana et al., 2002; Madrid et al., 2009; Abd-Elsayed et al., 2015). Moreover, two-pore potassium channels (K2P), such as TASK-3 and TRESK, have also been implicated in setting cold receptor activation thresholds and may also contribute to cold pain signaling (Castellanos et al., 2020; Hughes et al., 2017; Morenilla-Palao et al., 2014). Conversely, the roles of voltage-gated sodium channel subtypes (Na_V_1.1–1.9) in controlling cold receptor action potential firing and excitability remain less explored. In pathological conditions, such as peripheral neuropathies or spinal cord injury, cold signaling becomes impaired and a normally innocuous cold stimulus is perceived as painful, referred to as cold allodynia (Jensen and Finnerup, 2014; Yin et al., 2015; MacDonald et al., 2020; Shiao and Lee-Kubli, 2018). The extent to which physiological cold sensing and cold pain share common signaling mechanisms is unclear. Importantly, chronic pain affects 50.2 million adults in the U.S. (Yong et al., 2022) with an estimated prevalence of 10% of the global population (Zimmer et al., 2022; Jackson et al., 2014). Although cold allodynia is one of the most common complaints (MacDonald et al., 2020), there are very few successful treatments. A comprehensive understanding of the ion channels that give rise to this condition, as well as those that contribute to normal cold sensing, could aid in identifying putative molecular targets for the rational development of drugs to treat cold allodynia (Esposito et al., 2019).

In this Review, we will hone in on the specific ion channels associated with the transduction and transmission mechanisms of cold sensation by peripheral sensory neurons in both health and disease. Integration of this information with other work discussing cold encoding (Lolignier et al., 2016; McKemy, 2018; Buijs and McNaughton, 2020; MacDonald et al., 2020; Lewis and Griffith, 2022) will advance our basic understanding of cold signaling.

## Cold transduction mechanisms

### TRP channels as cold transducers

**Figure 1.**
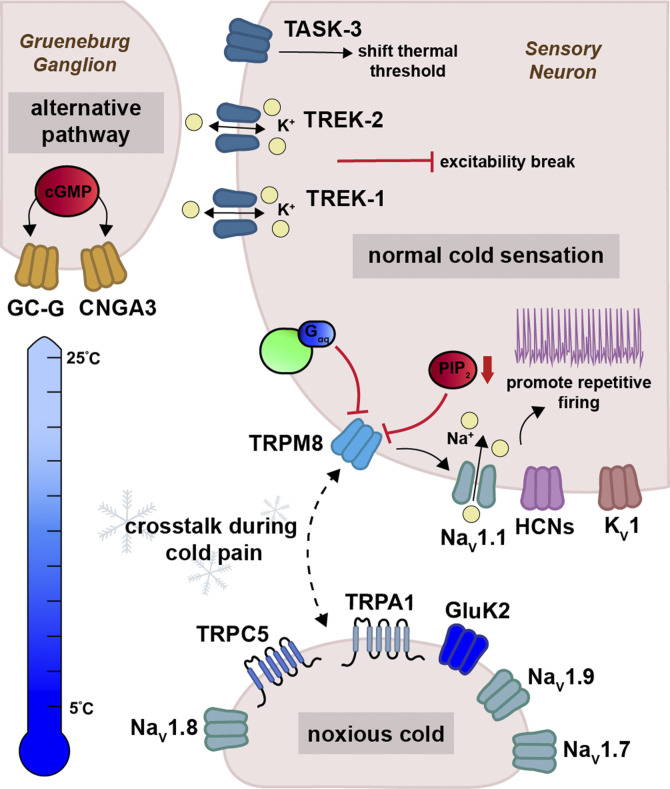
**Ion channels of cold transduction and transmission.** Diverse collections of ion channels function in concert to transmit innocuous and noxious cold information. Under normal physiological conditions, the transduction of cold signals is aided by the activation of TRP channels such as TRPM8, TRPA1, and TRPC5, which likely engage in functional crosstalk during cold pain. GluK2 may represent a new cold transduction channel specifically tuned to detect noxious cold temperatures (i.e., below 10°C). Grueneberg ganglion (GG) neurons of the olfactory system lack TRPM8 and thus utilize alternative transduction mechanisms. Cold-sensing GG neurons require cGMP signaling to activate the cyclic nucleotide-gated (CNG) channel CNGA3 and the Guanylyl cyclase enzyme GC-G. Cold transmission, on the other hand, requires the activation of various voltage-gated sodium and potassium channels whose modulation is responsible for the perception of cold stimuli either through engaging excitability breaks, as seen with TREK channels TREK-1 and TREK-2 or by promoting repetitive firing like HCN channels and the Na_V_1.1 voltage-gated sodium channel.

Mammalian cold-sensing neurons express a diverse array of ion channels and receptors responsible for detecting reductions in temperature of <1°C (Fig. 1). TRPM8 was the first cold transduction channel to be cloned (McKemy et al., 2002; Peier et al., 2002) and is expressed in cold-sensitive sensory neurons, as well as in prostate epithelium (Bidaux et al., 2015; Ramachandran et al., 2013; Asuthkar et al., 2015), sperm (De Blas et al., 2009), and thermoregulatory circuits (Ordás et al., 2021). TRPM8 is found in 10–15% of small diameter sensory neurons in both TG and DRG, innervating tissues such as the tongue, skin, teeth, and the cornea (Abe et al., 2006; Dhaka et al., 2008; Jankowski et al., 2017; Tsutsumi et al., 2010; Parra et al., 2010). In addition to cold, TRPM8 is activated by chemical compounds such as menthol and icilin, membrane depolarization, and several inflammatory agents (McKemy et al., 2002; Rohács et al., 2005; Voets et al., 2007; Brauchi et al., 2006; Linte et al., 2007; Voets et al., 2004).

Each TRPM8 protomer contains a large cytoplasmic N-terminal domain (NTD) comprising four melastatin homology regions (MHR1–4) and a pre-MHR region (Yin et al., 2018; Palchevskyi et al., 2023). The transmembrane domain then follows with six transmembrane helices composed of a pre-S1 region, a voltage-sensor-like domain (VSLD), and a pore. This transmembrane region is then followed by a TRP helix and the C-terminal domain (CTD) containing two connecting helices and a coiled-coil domain (Yin et al., 2018, 2022; Enklaar et al., 2000; Diver et al., 2019). Interestingly, it is thought that during evolution, as tetrapods transitioned from water to land, the accumulation of mutations in the MHR1–3 domain resulted in the cold sensitivity of TRPM8 proteins (Lu et al., 2022). The TRPM8 CTD is formed by neutral, polar, and hydrophobic amino acids (Yin et al., 2018) and is required for the temperature-driven gating of TRP channels (Brauchi et al., 2006; Díaz-Franulic et al., 2020). TRPM8 is highly thermosensitive, with a calculated Q_10_ value of ∼24 (Raddatz et al., 2014; Brauchi et al., 2004). Upon deletion of the last 36 amino acids of the CTD, the Q_10_ is reduced to 4, highlighting the necessity of this structure for the temperature sensitivity of TRPM8 with the folding-unfolding reaction of the CTD likely dictating the sensitivity of TRPM8 to cold through an increase in molar heat capacity (Díaz-Franulic et al., 2020). In addition to temperature, chemical agonists like menthol act as gating modifiers to prime the channel for activation by shifting the voltage dependence of TRPM8 toward physiological membrane potentials and shifting the temperature threshold to higher temperatures (Voets et al., 2004; Mälkiä et al., 2007).

The cryo-EM structure of the full-length channel gave additional insight into the polymodal nature of TRPM8 gating (Zhao et al., 2022). The pore contains the binding sites for calcium and other ligands while the VLSD is thought to contain a menthol binding site in its cavity. Interestingly, unlike TRPV channels, the S4 of the VLSD contains arginine residues that are responsible for its voltage dependence and have been reported to play a role in its activation by cold and menthol (Voets et al., 2007; Raddatz et al., 2014; Yin et al., 2018; Díaz-Franulic et al., 2020). TRPM8 activation requires PIP_2_ signaling to sensitize the channel (Rohács et al., 2005; Liu and Qin, 2005; Daniels et al., 2009; Brenner et al., 2014). PIP_2_ binding affinity differs between open, closed, and intermediate states, with large state-dependent conformational changes upon ligand and PIP_2_ binding (Yin et al., 2022). Conversely, increased levels of PIP_2_ result in channel activation at warmer temperatures (Rohács et al., 2005). Several residues in the TRP domain, pre-S1 segment, and N-terminus have been identified as sites for this regulation (Yin et al., 2018), and structural analyses have identified small changes in the binding site of the VLSD that trigger conformational changes for gating (Yin et al., 2022). TRPM8 function is also regulated by inflammatory mediators such as bradykinin and nerve growth factor via activation of G-protein coupled receptors (GPCRs) that stimulate phospholipase C, which was originally thought to downregulate TRPM8 function through activation of protein kinase C (Babes, 2009; Premkumar et al., 2005; Abe et al., 2006). However, a more recent study found that Gα_q_ proteins bind to three arginine residues in the N-terminal of TRPM8, and mutation of these residues abolished the inhibitory effect of the bradykinin on TRPM8 channel activity, although sensitivity to PIP_2_ remained intact (Zhang, 2019). Furthermore, this study showed that the bradykinin receptor BR2 binds to TRPM8, which renders the channel insensitive to PIP_2_ depletion. Thus, complex signaling crosstalk induced by inflammatory mediators and their receptors regulates TRPM8 activity.

Similar to TRPM8, transient receptor potential ankyrin 1 (TRPA1) is a polymodal cation channel and is primarily activated by a wide range of noxious ligands (Bautista et al., 2006; Jordt et al., 2004; Nagatomo and Kubo, 2008) as well as noxious temperatures. It is primarily expressed in peptidergic C-fibers and plays a major role in the transduction of nociceptive signals linked to tissue damage, inflammation, and neuropathic pain (Laursen et al., 2014). It was first cloned in 1999 in cultured human fibroblasts (Jaquemar et al., 1999) and is the only mammalian member of the TRPA family. Its role in cold sensing, however, was not discovered until 2003 (Story et al., 2003). Cool temperatures shift the voltage dependence of activation of TRPA1 toward more negative potentials, reduce the temperature sensitivity of the rate of deactivation, and increase open probability (Karashima et al., 2009). Like TRPM8, menthol activates TRPA1, but at concentrations over 1 mM, menthol will reversibly block the channel. However, whether menthol directly activates TRPA1, or the channel is indirectly activated by cold-induced calcium influx, remains unclear (Yamaguchi et al., 2023; Zurborg et al., 2007). Despite initially being characterized as a cold-sensing channel, TRPA1 has since become controversial for its role in cold sensation in vivo. At the behavioral level, loss of TRPA1 only results in partial deficits in cold responses, if any at all (Bautista et al., 2006; Karashima et al., 2009; Brenner et al., 2014) or has a minor sex-dependent effect (Kwan et al., 2006). It is possible that TRPA1 synergizes with TRPM8 to encode for the entire cold temperature range, indicating that TRPA1 alone is insufficient to drive cold behavioral responses (Winter et al., 2017). In primates, TRPA1 is not activated by cold, suggesting that cold sensitivity of TRPA1 differs among mammalian species, with a single residue in the S5 domain thought to underly this difference (Chen et al., 2013). Indeed, in rodents, mutations of this residue abolish cold activation and alter the voltage-dependent characteristics of this channel (Zíma et al., 2015).

As a member of the TRP family, TRPA1 contains six transmembrane domains, a re-entrant pore loop, and large cytoplasmic CTD and NTD regions that account for roughly 80% of the channel’s overall mass (Paulsen et al., 2015). The NTD 14 ankyrin repeats, known as the ankyrin repeat domain (ARD), which are thought to be important for modulating gating and channel activation due to the presence of select binding sites (Talavera et al., 2020; Zayats et al., 2013). The linker region that connects neighboring ankyrin repeats serves as a binding site for potent compounds such as allyl isothiocyanate (horseradish, wasabi, and mustard), allicin (raw garlic), and cinnamaldehyde (cinnamon), with certain regions tied to thermal and chemical sensitivity (Cordero-Morales et al., 2011; Jabba et al., 2014). Surprisingly, TRPA1 remains both chemo- and cold-sensitive without its ARD (Moparthi et al., 2014), instead requiring the CTD allosterically coupled to the S5–S6 pore region and VSLD for heat and cold sensitivity, respectively (Moparthi et al., 2022). The ARD is among the longest of the vertebrate TRP channels, yet very little is currently known about its explicit function in cold sensation.

The transient receptor potential canonical 5 (TRPC5) has been identified as a cold sensor that works in conjunction with TRPA1 to detect cold in the teeth of mice (Bernal et al., 2021). Utilizing an ex vivo jaw-nerve preparation, both pharmacological inhibition and genetic deletion of TRPC5 significantly reduced cold responses. TRPC5 is cold-sensitive between 25 and 37°C and is directly activated by extracellular calcium levels and G-protein stimulation, with recent studies reporting that PIP_2_ both activates and desensitizes the channel through independent gating mechanisms (Nilius and Owsianik, 2011; Ningoo et al., 2021). Deletion of TRPC5 results in no temperature-sensitive behavioral changes but rather upregulates TRPM8 and other menthol-sensitive channels (Zimmermann et al., 2011). These findings suggest that the role of TRPC5 in cold sensation is limited to the detection and regional adaptation of cold temperatures.

### Alternative cold transduction mechanisms

In addition to temperature sensing in the skin and teeth, cold sensitivity plays an important role in other organs such as the tongue, eyes, and nose. In the olfactory system, cold-sensitive neurons of the Grueneberg ganglion (GG) function as unidirectional thermosensors and are finely tuned to detect small decreases in temperature within narrow temperature windows. Unlike other cool-sensitive cells, GG neurons lack TRPM8, suggesting other channels are responsible for detecting cold temperatures (Fleischer et al., 2009; Schmid et al., 2010). Notably, signaling within cold-sensitive GG neurons requires cGMP, which activates cyclic nucleotide-gated (CNG) channels and nonselective heterotetrameric cation channels formed by CNGA and CNGB subunits. CNG α subunit 3 (CNGA3) is strongly expressed in cold-sensitive GG neurons and is critical for both chemosensory and thermosensory signal transduction (Mamasuew et al., 2008; Bumbalo et al., 2017). In GG neurons, CNGA3 is activated by cyclic guanosine monophosphate (cGMP), a process potentiated by temperatures below 22°C. CNGA3 transcript has also been found in cold-sensitive DRG neurons (Luiz et al., 2019). More recently, CNGA3 was found to act as a cold sensor that regulates the cold responses of neurons in the thermoregulatory center of the hypothalamus of mice, illuminating a potentially novel physiological role of CNGA3 in cold sensitivity in central nervous system circuits (Feketa et al., 2020).

Guanylyl cyclases (GCs, GC-A to GC-G) are ubiquitously expressed enzymes that regulate various cellular processes. They consist of an extracellular domain, a short transmembrane region, and an intracellular C-terminal catalytic region (Kuhn, 2016). GC-G, like CNGA3, is found in GG neurons and is activated by cGMP, low levels of carbon dioxide, and cool temperatures (Liu et al., 2009; Sun et al., 2009; Chao et al., 2015). In the current working model of temperature sensing in GG neurons, cool temperatures trigger GC-G activity (Mamasuew et al., 2010), leading to the activation of CNGA3 (Bumbalo et al., 2017), and the subsequent closure of the leak potassium channel TREK-1, all contributing to a depolarization of the cell membrane (Fleischer, 2021; Stebe et al., 2014).

Most recently, the kainite receptor, GluK2, has been proposed to function as a noxious cold detector in the peripheral nervous system (Cai et al., 2024). Prior work identified the invertebrate glutamate receptor-like channel, GLR-3, in the *C. elegans* sensory neuron ASER as necessary for normal cold avoidance behaviors (Gong et al., 2019). The authors now show that the mammalian homolog of GLR-3, GluK2, is required for the detection of noxious cold temperatures and cold nociception in mice, potentially identifying a new noxious cold transduction channel. Interestingly, the authors proposed that GluK2’s role in cold transduction is not due to its ion channel function, but instead a noncanonical metabotropic mechanism.

## Cold transmission mechanisms

In 1999, Suto and Gotoh first observed increases in cellular Ca^2+^ concentration in cultured rat DRG neurons upon temperature reduction (Suto and Gotoh, 1999). Although it was initially thought that signaling mechanisms were due to the enzymatic function of the Na^+^/K^+^ ATPase, it was later found that both sodium and potassium currents drive cold transmission.

### Voltage-gated potassium channels

Within the voltage-gated potassium channel family (K_V_), K_V_1.1 and K_V_1.2 are notable for their major role in modulating the excitability of temperature-sensitive neurons (Madrid et al., 2009; Zhou et al., 1998; González et al., 2017). Opening of these channels produces a hyperpolarizing current called I_KD_ (Storm, 1988; Viana et al., 2002) that dampens the depolarizing effect of TRPM8-dependent currents by shifting the temperature thresholds of individual neurons to colder values and reducing their overall responsiveness (Madrid et al., 2009; Lolignier et al., 2016). In the presence of the potassium channel blocker 4-AP, cold sensitivity can be induced in normally cold-insensitive neurons, which is attributed to the inhibition of I_KD_ (Viana et al., 2002; MacDonald et al., 2021). This is in line with prior work showing that cells with high threshold cold responses have a larger I_KD_ (Madrid et al., 2009).

### Two-pore domain potassium channels

Two-pore domain potassium channels (K2P) channels are leak potassium channels that control neuronal excitability by tuning the resting membrane potential and are most often expressed in sensory neurons, cardiac muscle, skeletal muscle, retinal cells, and various brain regions (Aller and Wisden, 2008; Cadaveira-Mosquera et al., 2012; Grandi et al., 2017; Hughes et al., 2017; Weir et al., 2019; Herrera-Pérez et al., 2021; Luo et al., 2021). Despite their title as leak channels, K2P channels are not permanently open. Instead, K2P channel’s open probability increases upon depolarization and their gating is dependent on time and voltage, despite lacking a specialized voltage-sensing domain (Bockenhauer et al., 2001; Schewe et al., 2016). TREK-1, TREK-2, and TRAAK channels are mechanothermal K2P channels whose function is modulated by interactions with lipids (Brohawn et al., 2014; Riel et al., 2022). These channels control both warm and cold perceptions, and in DRG, are most abundantly expressed in small-diameter neurons (Viatchenko-Karpinski et al., 2018). Electrophysiological analyses show large leak potassium currents at 22°C, which are inhibited at cool temperatures around 14°C and potentiated by temperatures around 30°C and higher (Viatchenko-Karpinski et al., 2018).

TREK-1 and TREK-2, unlike other K2P channels, are generally not active at room temperature, but activate in response to stretch, intracellular pH, and heat (Patel et al., 1998; Maingret et al., 1999; Kim et al., 2001). TREK-1, the second K2P channel to be cloned (Fink et al., 1996), is important in controlling cell excitability, and shifts from being voltage-dependent to more “leak-like” when the open probability is increased through stretching of the membrane, intracellular acidosis or PIP_2_ stimulation, or mutation of its proton sensor (Maingret et al., 1999; Chemin et al., 2005; Sandoz et al., 2009; Honoré, 2007). TREK-1 colocalizes with TRPV1 in nociceptors where it functions to detect noxious temperatures as well as painful mechanical stimulation (Alloui et al., 2006; Noël et al., 2009). TREK-1 activation is associated with tooth pain (Magloire et al., 2003; Hermanstyne et al., 2008) and aids in migraine alleviation (Ávalos Prado et al., 2021). TREK-1 and TRAAK activity is decreased at cold temperatures below 17°C, and for noxious cold detection, they function to silence heat-nociceptors (Noël et al., 2009). While deletion of TREK-1 does not affect the overall percentage of cold-sensitive DRG neurons, DRG from TREK-1^−/−^;TRAAK^−/−^ double knockout mice display a significant increase in cold-sensitive DRG neurons, and at the behavioral level, mice display increased sensitivity to cool temperatures, suggesting that these channels work in concert during cold sensing in vivo (Noël et al., 2009).

K2P channels of the TASK clade are pH-sensitive, activating in response to changes in acidity even within a physiological range (7.2–7.3). Of the TASK family members, TASK-3 is the least pH-sensitive, activating at pH levels between 6.0 and 6.7. TASK-3 is primarily expressed in small-diameter sensory neurons and colocalizes with TRPM8 and TRPV1. Intriguingly, it is enriched ∼140-fold in TRPM8-positive neurons. Genetic deletion of TASK-3 results in the loss of high-threshold TRPM8-expressing cold neurons, thus rendering mice more sensitive to cold stimuli (Morenilla-Palao et al., 2014), demonstrating that normal cold sensing involves a delicate balance of excitatory and inhibitory currents for appropriate cold sensitivity.

### HCN channels

The hyperpolarization-activated cyclic nucleotide-gated (HCN) ion channel family is expressed in both the central and peripheral nervous system and is responsible for the hyperpolarization-activated current or H-current (I_h_). HCN1 and HCN2 are the isoforms most strongly associated with primary somatosensory neurons and are thought to control the excitability of cold receptors; however, I_h_ is not required for the transduction of cold stimuli in cold-sensitive neurons of the trigeminal ganglia (Orio et al., 2009). Instead, it is thought to play a role in cold transmission or encoding. Indeed, in HCN1 null mice, I_h_ of trigeminal cold-sensing neurons was nearly abolished and resulted in suppressed cold sensitivity following exposure to the cold plate (Orio et al., 2009).

### Voltage-gated sodium channels

Five of the nine mammalian voltage-gated sodium channel (Na_V_) isoforms are expressed in healthy adult sensory neurons: Na_V_1.1, Na_V_1.6, Na_V_1.7, Na_V_1.8, and Na_V_1.9. Currently, very little is known regarding the specific isoforms that transmit cold signals under physiological conditions. Originally, Na_V_1.8 was proposed to transmit information at noxious cold temperatures (Zimmermann et al., 2007). While the steady-state inactivation curves of tetrodotoxin-sensitive Na_V_s, like Na_V_1.7, were found to shift to more hyperpolarizing potentials at noxious cold temperatures, Na_V_1.8 function was unaltered by cold. However, in vivo calcium imaging found that Na_V_1.8 was not required for sensory neuron responsiveness to cold, even at noxious temperatures (Luiz et al., 2019). More recently, Na_V_1.1 was found to drive firing in murine TRPM8-expressing DRG neurons in vitro (Griffith et al, 2019). In these neurons, Na_V_1.1 channels were proposed to enable repetitive firing by quickly cycling through fast-inactivated states while circumventing long-lived slow-inactivated states. Whether Na_V_1.1 is required for cold sensing in vivo remains to be determined. Recent findings indicate that generally, the gating of Na_V_s is temperature-sensitive where the voltage dependence of activation hyperpolarizes as the temperature increases (Kriegeskorte et al., 2023).

## Ion channels of noxious cold signaling

A subpopulation of cold-sensitive neurons is also sensitive to capsaicin, suggesting that some cold-sensing neurons are polymodal nociceptors that express TRPV1 in addition to TRPM8 (Viana et al., 2002; Reid and Flonta, 2002; McKemy et al., 2002; Xing et al., 2006; Hjerling-Leffler et al., 2007). However, our understanding of the mechanistic differences between noxious cold sensing and cold-induced pain is murky. Noxious cold signaling is essential for detecting stimuli that typically fall below 12°C and results in the activation of cold nociceptors (Morin and Bushnell, 1998; Knowlton et al., 2013). Alternatively, cold allodynia and cold hyperalgesia, in which innocuous cold is perceived as painful or when noxious cold produces a more pronounced pain response, respectively, are common complaints of those suffering from peripheral neuropathy (Finnerup et al., 2021). It is unclear to what extent discrete ion channels differentially contribute to these pathways, or if shared mechanisms exist.

TRPM8, in addition to being well known for its role in innocuous cold sensing, has been shown to respond to temperatures in the noxious range in vitro (McKemy et al., 2002), and in some cases, mice lacking TRPM8 exhibit defective responses to noxious cold (Bautista et al., 2007; Colburn et al., 2007; Dhaka et al., 2007; Knowlton et al., 2010). Interestingly, this phenotype is not found in other studies which show that noxious cold avoidance is preserved in TRPM8-null mice (Knowlton et al., 2013; Pogorzala et al., 2013). Double knockout of TRPM8 and TRPA1 in mice has also produced conflicting results, where cold avoidance is either the same (Knowlton et al., 2010) or exacerbated (Winter et al., 2017) compared with TRPM8 null mice. More recently, it was reported that blocking the transmission of TRPA1-positive afferents can inhibit noxious cold behaviors (Yamaki et al., 2021). The working model posits that the release of artemin, a neurotrophic factor, from TRPA1-expressing terminals activates and sensitizes TRPM8 channels via GFRα3 signaling, ultimately resulting in the development of cold allodynia and hyperalgesia (Lippoldt et al., 2016). Indeed, further exploration revealed that inflammatory mediators released by TRPA1 and TRPV1 activation result in cold allodynia through activation of the CGRP receptors NK1R and TLR4 via localized artemin release (Yang et al., 2023). This further supports the idea that TRPM8 and TRPA1 work in conjunction to transmit noxious cold signals.

In non-mammalian species, TRPA1 functions as a heat sensor (Laursen et al., 2015; Cordero-Morales et al., 2011), and in 2016, human TRPA1 was found to be a bidirectional thermosensor, providing evidence that the channel’s inherent heat sensitivity is evolutionarily conserved. TRPA1 activates below 17°C (a temperature considered in the painful range for humans) (Story et al., 2003) and above 30°C (Moparthi et al., 2016), with both cold and heat sensitivity influenced by the channel redox state and ligand partners. Interestingly, triple knockout of TRPA1, TRPM3, and TRPV1 in mice eliminates the detection of noxious heat while maintaining responses to both noxious cold and mechanical stimuli (Vandewauw et al., 2018). However, acute heat responsiveness remains when at least one of the three channels is functional, suggesting the presence of fail-safe mechanisms for the avoidance of life-threatening heat exposure. TRPA1 likely takes part in maintaining noxious temperature detection on both ends of the spectrum to ensure survival in circumstances in which other major thermosensors become non-functional. Currently, it is unclear whether other TRP channels function similarly to TRPA1 with U-shaped thermosensitivities.

Sodium channels and various sodium channel mutations are associated with an array of pain disorders (Cox et al., 2006; Wu et al., 2013; Huang et al., 2014; Han et al., 2015, 2017; Leipold et al., 2015; Devor, 2006; Bennett et al., 2019). Loss of Na_V_1.9 results in cold insensitivity phenotypes (Lolignier et al., 2015), while gain-of-function mutations lead to cold-aggravated pain (Leipold et al., 2015). These findings highlight the importance of Na_V_1.9 in regulating noxious cold detection in both normal and pathological conditions. Loss of function mutations of Na_V_1.7 have been closely linked to pain syndromes and heightened sensitivity to heat (Minett et al., 2014; Yang et al., 2016; Kriegeskorte et al., 2023). As discussed above, the role of Na_V_1.8 in noxious cold signaling is less clear. It was proposed to be the sole Na_V_ isoform responsible for action potential generation in nociceptors at cold temperatures (Zimmermann et al., 2007). Additionally, the loss of Na_V_1.8-positive neurons resulted in impaired nocifensive behavior in response to a −5°C cold plate, suggesting that Na_V_1.8 is important for detecting extreme cold temperatures (Luiz et al., 2019). Loss of Na_V_1.8 has also been shown to attenuate oxaliplatin-induced cold allodynia (MacDonald et al., 2021). Future studies are needed to settle the debated role of this channel in normal and noxious cold sensing, as well as cold-induced pain.

### Cold-induced pain

#### Therapeutic targets for cold pain

Recent studies suggest that biomarkers for cold sensitivity may serve as diagnostic tools for more individualized pain therapies. L-menthol, for example, has been used topically to recapitulate cold allodynia in healthy subjects by sensitizing cold-sensitive afferents (Andersen et al., 2014). However, some subjects reported unexpected symptoms ranging from the feeling of warmth to the development of spontaneous pain (Hatem et al., 2006; Binder et al., 2011; Wasner et al., 2004), highlighting crosstalk between cold and mechanical pain circuits. Paradoxically, extended exposure to L-menthol has been shown to desensitize cold-sensitive fibers and c-nociceptor fibers, acting as an analgesic (Wasner et al., 2004). Human surrogate models like L-menthol will be important for screening and testing novel therapies and treatments.

Currently, opioids are first-order drugs used to treat severe pain, but due to substantial side effects and the high risk of developing opioid use disorder, alternative management strategies are required. In recent years, selective α9α10 nicotinic acetylcholine receptor antagonists (such as RglA4) have been highlighted for their success in treating chemotherapy-induced neuropathic pain and attenuating cold allodynia in mice and having high potency for human nAChRs (Romero et al., 2017; Huynh et al., 2022; Hone et al., 2018; Hone and McIntosh, 2023). Considering alterations in neuronal conductance are observed across several models of cold allodynia, targeting ion channel dysregulation may be important in reducing afferent hyperexcitability. This has been supported by previous clinical trials in which some non-specific sodium channel inhibitors have been efficient in treating cold pain in patients suffering from diabetic neuropathy (Eisenberg et al., 1998); however, more recent studies have reported inconsistencies in the efficacy of more targeted sodium-channel blockers for pain disorders (Siebenga et al., 2020; McDonnell et al., 2018; Dormer et al., 2023). These findings highlight how the major challenge with pharmacology is ensuring cell-type-specific targeting while preserving analgesic effects. Additionally, a spotlight has been placed on both TRPM8 and TRPA1 modulators as potential treatments for cold allodynia. Compound 8, also known as PF-05105679—a TRPM8 inhibitor—has been optimized for efficacy and has been shown to reduce cold pain in humans (Winchester et al., 2014; Andrews et al., 2015). More recently, Aconitine, a TRPA1 antagonist, has been shown to alleviate cold and mechanical allodynia in mice with cancer-induced bone pain (Jin et al., 2023). Finally, Sigma1 receptor antagonists, which inhibit TRPA1 plasma membrane trafficking and function, have been shown to reduce and prevent behavioral symptoms of cold and mechanical hypersensitivity in mice using the oxaliplatin-induced peripheral neuropathy model (Marcotti et al., 2023).

## Concluding remarks

TRP channels, voltage-gated sodium and potassium channels, as well as an array of other ion channel types, such as ionotropic glutamate receptors, have been shown to transduce and transmit cold signals, sometimes in a cell-type-specific manner. Nevertheless, there are still major gaps in knowledge regarding the exact channels that mediate cold sensation in vivo and the precise mechanisms and pathways through which they do so. Despite major advances in our understanding of normal cold sensing, more work is required to tease apart the distinct, or shared pathways responsible for cold signaling, noxious cold, and cold pain. As we continue to develop technological methods to better parse apart major players, it has become clear that mechanisms underlying these conditions can be etiology-specific (Draxler et al., 2014). Thus, understanding the molecular, cellular, and physiological mechanisms that distinctly mediate physiological and noxious cold sensing from cold pain will be imperative for developing successful therapies and unraveling the distinct roles ion channels play in overall cold sensing.
